# BrdU Pulse Labelling *In Vivo* to Characterise Cell Proliferation during Regeneration and Repair following Injury to the Airway Wall in Sheep

**DOI:** 10.1155/2013/871932

**Published:** 2013-02-28

**Authors:** B. Yahaya, G. McLachlan, D. D. S. Collie

**Affiliations:** ^1^Cluster for Regenerative Medicine, Advanced Medical & Dental Institute (AMDI), Universiti Sains Malaysia, No. 6, Level 1 (Lot 13), Persiaran Seksyen 4/9, 13200 Kepala Batas, Penang, Malaysia; ^2^Easter Bush Veterinary Centre and The Royal (Dick) School of Veterinary Studies, The Roslin Institute, The University of Edinburgh, Roslin, Midlothian, Edinburgh EH25 9RG, UK

## Abstract

The response of S-phase cells labelled with bromodeoxyuridine (BrdU) in sheep airways undergoing repair in response to endobronchial brush biopsy was investigated in this study. Separate sites within the airway tree of anaesthetised sheep were biopsied at intervals prior to pulse labelling with BrdU, which was administered one hour prior to euthanasia. Both brushed and spatially disparate unbrushed (control) sites were carefully mapped, dissected, and processed to facilitate histological analysis of BrdU labelling. Our study indicated that the number and location of BrdU-labelled cells varied according to the age of the repairing injury. There was little evidence of cell proliferation in either control airway tissues or airway tissues examined six hours after injury. However, by days 1 and 3, BrdU-labelled cells were increased in number in the airway wall, both at the damaged site and in the regions flanking either side of the injury. Thereafter, cell proliferative activity largely declined by day 7 after injury, when consistent evidence of remodelling in the airway wall could be appreciated. This study successfully demonstrated the effectiveness of *in vivo* pulse labelling in tracking cell proliferation during repair which has a potential value in exploring the therapeutic utility of stem cell approaches in relevant lung disease models.

## 1. Introduction

The lung is a relatively stable organ with low rates of cell turnover, particularly, in the airways where less than 5% of epithelial cells proliferate at any given point in time [1]. There appears to be a little need for local self-renewal under normal circumstances. However, in the presence of an acute epithelial injury, it is recognised that cell proliferation is a notable feature of repair responses. Evidence from studies in small animals suggests that following physical injury the cells bordering the lesion dedifferentiate, flatten, proliferate, and migrate over the denuded area to restore the barrier function of the epithelium [2–8]. The key to understanding the factors that divert repair away from resolution and towards remodelling is a close understanding of these dynamic events that operate to resolve epithelial injury. However, as species differences exist in relation to the structure and composition of the airway wall, it is reasonable to speculate that similar variation in the temporal dynamics of the repair process may also exist between species. In order to bridge the gap between studies in small animals and man, a gap assumed on the basis of anatomical and physiological contrasts, we sought to characterise the airway wall repair response in sheep, a species of acknowledged relevance as a model for several lung diseases [9–12]. Such approach carries the additional advantage of potentially aligning perturbation and assay techniques with those relevant to clinical and veterinary clinical disciplines. Whilst we have recently published data in which anti-Ki-67, a nuclear protein associated with cell proliferation, antibody was used to characterise aspects of the proliferative response in this ovine model [13], we recognise that the use of BrdU in the setting of *in vivo* administration might offer advantages to identify slowly proliferating niches of such cells in the airway wall. Further, the known sensitivity of such labelled cells to UV light [14] might offer utility in being able to selectively target label-retaining cells *in vivo* and investigate the consequence in relation to wound repair. As such, it was necessary to ascertain whether the proliferative cell response characterised using BrdU was qualitatively consistent with that previously characterised by our group using this model.

## 2. Material and Method

### 2.1. Animal

All experimental procedures were subjected to ethical review at the University of Edinburgh and were performed under licence, as specified by the Animals (Scientific Procedures) Act 1986. Nine commercially sourced crossbred sheep aged between 14 and 16 months were used in this study (body weight 59.4 ± 8.8 kg). 

### 2.2. Brushing Procedure

Anaesthesia was induced by intravenous injection of thiopentone sodium (20 mg/kg bodyweight, Thiovit, Novartis, UK) and, thereafter, maintained by 2% Isophane in nitrogen oxide (NO) and oxygen (O_2_) (50 : 50) for the duration of the brushing protocol (15–30 sec/brushing). Sheep were ventilated by positive pressure ventilation (Harvard Apparatus Model 708 (MA, USA)) with tidal volume set at 10 mL/kg body weight. Brushing procedures carried out in this study were performed as previously described [13]. One hour before the sheep were killed, BrdU was infused via the jugular vein at a dose of 10 mg/kg body weight diluted to 10 mg/mL in sterile phosphate buffered saline (PBS) solution. Each sheep was euthanised one hour after BrdU infusion by intravenous injection of sodium pentobarbital (Euthatal, Merial Animal Health Ltd., Essex, UK). During the postmortem, both brushed and nonbrushed (opposite to the brushed area and naïve control) sites were carefully identified and dissected away from the surrounding lung tissue. 

### 2.3. Tissue Processing and Staining

Tissues were fixed with 10% neutral buffered formalin for at least 12 hours before being dehydrated in alcohol and embedded in paraffin. Cut tissue sections (5 *μ*m) were fixed on adhesive slides (X-Tra, Surgipath, Peterborough, UK) and glass slides and incubated overnight at 37°C. Tissue sections were subjected to standard haematoxylin and eosin (HE) and immunohistochemical (IHC) staining of BrdU (acid denatured with 2 M hydrochloric acid (HCl) for 8 minutes in 60°C followed by treatment with trypsin (pH 7.8) for 30 minutes at 37°C was used for antigen retrieval steps). Subsequently, tissue sections were incubated overnight with mouse anti-BrdU (1 : 500 dilutions) antibody at 4°C. Visualisation protocols were as specified by the manufacturer (RTU Vectastain Universal Elite ABC commercial kit, Vector Laboratories, Peterborough, UK). Negative control sections, processed and stained as described, were either derived from an animal not exposed to BrdU or from BrdU-treated animals, but with the primary antibody step omitted. The numbers of cells with BrdU positive-nuclei within each area of interest (damaged, transitional, and undamaged) were directly counted and record of their distribution within tissue compartments (mucosal or submucosal) was kept. For absolute cell count data, the Poisson model was used to investigate differences in the total number of positive cells between compartments, areas of interest, and time points. The data were analysed using the freely available statistical software environment R (The R Foundation, Software version 2.9.0, Vienna, Austria) as previously described [13]. In all cases, a *P* value of ≤0.05 was considered statistically significant.

## 3. Result

**Figure 1:**
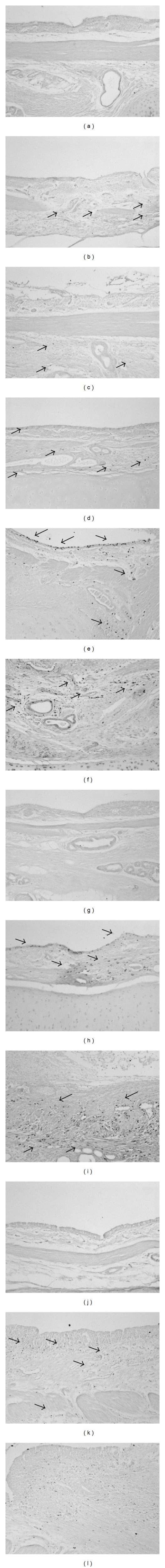
Airway tissue sections stained with BrdU. Histological images (×10) of airway wall sections from airways undergoing repair 6 hours ((a)–(c)), 1 day ((d)–(f)), 3 days ((g)–(i)), and 7 days ((j)–(l)) after physical injury induced by bronchial brush biopsy. The leftmost column depicts the undamaged airway wall opposite the site of injury, the middle column: the transitional zone between that area, and the area directly damaged by the brush biopsy in the rightmost column. No increase in proliferative activity was apparent 6 hours after physical injury ((a)–(c)). By day 1, there was a noticeable increase in the proliferative activity in the epithelium of the transitional zone bordering the lesion (e) as well as in the mucosa and submucosa underlying the area of damage (f). By day 3, there was still evidence of cell proliferation in both the lesion margins (h) as well as in the organising matrix of the damaged wall (i). By day 7, the extent of proliferation had started to decline in both areas ((k), (l)).

Time-dependent changes were appreciated at the histological level. In general, the brushed sites were compared to the normal morphology of the airway (naïve and undamaged sites). In relation to proliferative activity in response to injury, the specific location and number of proliferating cells varied with time of injury where the proliferating cells were easily identified by their dark-stained nuclei. The numbers of BrdU-positive cells were dependent on the time points and locations (damaged and transitional versus undamaged areas mucosa versus submucosal regions) (Figure 1). By day 1, after injury, BrdU-labelled cells were visible within the airway epithelium in clusters flanking the lesion, that is, in the transitional regions and underneath the damaged epithelium in the mucosa and submucosa particularly in association with the submucosal glands (SMGs). At day 3, after injury, the BrdU-labelled cells that were present in the underlying mucosa included those with morphology consistent with fibroblasts and endothelial cells. By day 7, after injury, the epithelium at the brushing site included areas of stratified squamous epithelial cells consistent with squamous metaplasia. 

**Figure 2:**
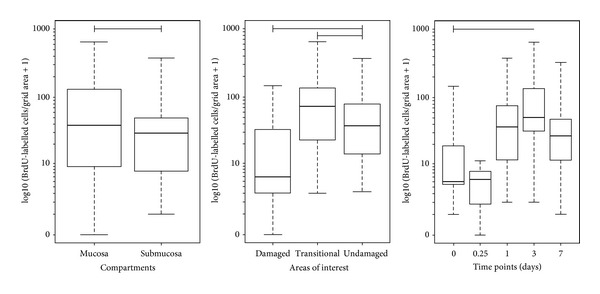
Distribution of BrdU-positive cells. Boxplot illustrating the numbers of BrdU cells counted in the airway tissues, separated by compartment, area of interest and time points. Overall cell counts were significantly greater for the mucosal versus submucosal (*P* < 0.001) compartments and for the damaged and transitional relative to the undamaged areas of interest. There was an increase in the numbers of BrdU-positive cells at day 3 post injury as compared to the naïve (time point 0) (*P* = 0.004). Upper and lower box plot margins represent the interquartile range; middle bar indicates the median. The points outside the ends of the whiskers are outliers. The graph was plotted based on the absolute number of cells stained with BrdU on each area measured. Arrows show BrdU-positive cells.

Statistical analysis (Figure 2) showed that there were significantly increased numbers of BrdU-positive cells in damaged (*P* < 0.001; geometric means = 62.439) and transitional (*P* = 0.001; geometric means = 35.450) areas as compared to undamaged (geometric means = 9.236) areas. Although there was evidence of mucosal loss and epithelial attenuation at both sites bordering the lesion at the 6-hour time point, the only evidence of BrdU-labelled cells was sparsely scattered through the mucosa and submucosa. From day 1 to day 3 after injuries, although there were large numbers of BrdU-positive cells observed at these time points, only at day 3 after injury, there was a demonstrable significant difference (*P* = 0.004; geometric means = 55.013) as compared to day 0 (naïve; geometric mean = 9.277). 

## 4. Discussion

Although BrdU pulse labelling was previously used in sheep to assess cell proliferation in wool follicles [15], to our knowledge, there has been no prior report using this technique to identify proliferating cells in the airway wall. The time-dependent changes in BrdU-expression in this model proved to be qualitatively consistent with our previous findings where Ki-67 positivity was used as our proliferative marker [13]. Whilst statistical consistency between the results of these separate studies was not absolute, any small differences were most likely attributable to the small sample sizes used in both. What was noticeable was that the numbers of BrdU-positive cells were consistently lower than Ki-67-positive cells, an observation that is in line with the incorporation of BrdU during S-phase only, whereas Ki-67 will stain throughout proliferation (G_0_ excepted). 

In both studies, we observed that the repair process involves neighbouring cell populations not directly overlying the area exposed to the injury; therefore, these BrdU-labelled cells might be transit-amplifying cells derived from resident progenitor cells that are proliferating in response to injury. This phenomenon was clearly described in previous reports [2, 7, 8] in which the early repair events involve cells spreading and migrating from the lesion borders into the denuded area. 

Interestingly, in this transition area where we see a clustering of BrdU-labelled cells at day 1, we have previously demonstrated that there is almost a complete loss of the normally abundant goblet cell population in addition to the remodelling of the epithelium [13]. We can only speculate whether the proliferating cells are actually derived from goblet or ciliated cells that have dedifferentiated into a form of airway “progenitor” cell. 

There was a noticeable proliferative activity associated with the submucosal glands underlying the wound, including proliferation within the epithelial lining cells.

## 5. Conclusion

In conclusion, this study demonstrates the effectiveness of a technique to *in vivo* label proliferating cells during the process of airway repair. Future studies should be carried out to characterise the type of cells that are predominantly involved in the airway epithelium regeneration and repair especially at day 3 after injury for cellular therapeutic target in the airway-related diseases.
